# Urate metabolism in the gut

**DOI:** 10.1093/lifemeta/loaf041

**Published:** 2025-11-22

**Authors:** Riccardo Percudani

**Affiliations:** Department of Chemistry, Life Sciences and Environmental Sustainability, University of Parma, Parma 43124, Italy

**Figure 1:**
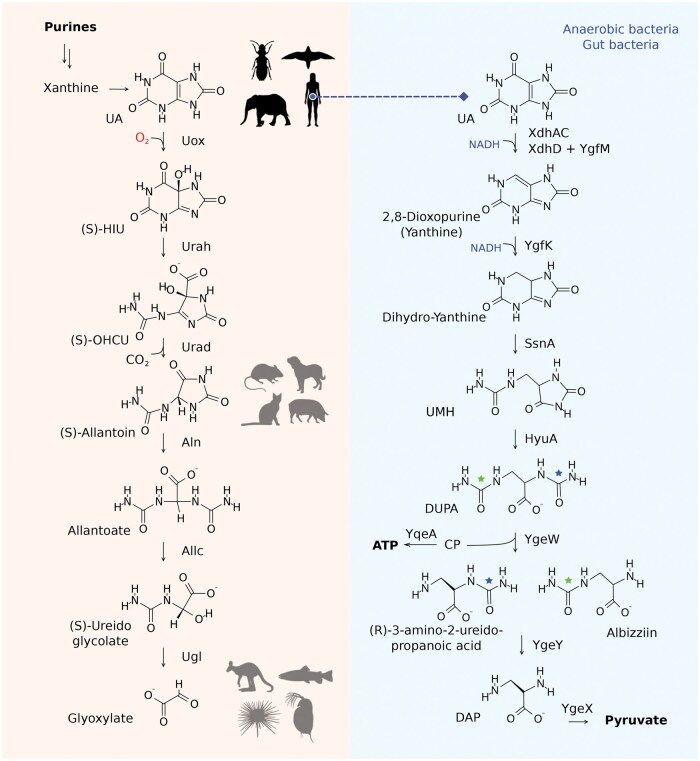
Comparison of the aerobic and anaerobic urate degradation pathways. Left: the animal oxidative pathway [6]. The final product of purine ­catabolism varies among species, as illustrated by representative silhouettes (phylopic.org). Right: the anaerobic reductive pathway characterized in gut bacteria [1, 2]. The two proposed variants differ in the ureido-group cleavage sequence: one involves first the 3-ureido group (green star), while the other involves first the 2-ureido group (blue star). Some reaction components are omitted for clarity. Abbreviations: CP, carbamoyl phosphate; DAP, 2,3-diaminopropanoic acid; DUPA, 2,3-diureido propanoic acid; HIU, hydroxyisourate; OHCU, 2-oxo-4-hydroxy-4-carboxy-5-ureidoimidazoline; UA, uric acid; UMH, ureidomethyl-hydantoin.

**Since Franz Schardinger first identified xanthine dehydrogenase (XDH) 123 years ago, the oxidation of purines to uric acid via the XDH pathway has been recognized as a central route in purine metabolism. Two recent studies now describe a previously unknown anaerobic uric acid degradation pathway in gut bacteria, highlighting its contribution to systemic urate homeostasis and its therapeutic promise for hyperuricemia and gout.** 

In contrast to most mammals, humans are unable to convert uric acid (UA), a purine derivative, into more soluble metabolites. Yet, it has long been known that this capacity resides in bacteria inhabiting our gut. The molecular players of this alternative route for UA degradation have been identified only recently, through the independent elucidation of a complete reductive pathway by two elegant studies [1, 2]. Amazingly, the enzymes and metabolites of the anaerobic pathway are completely different from the aerobic one (Fig. 1). This discovery is relevant to human health as the intestinal excretion of UA and its consumption by gut bacteria through the anaerobic pathway aid the elimination of a compound causing hyperuricemia and gout in humans [3, 4, 5].

The anaerobic degradation pathway starts, as expected, with the reduction of UA instead of its oxidation. However, unexpectedly, UA is not reduced to its precursor xanthine (2,6-dioxopurine) but to 2,8-dioxopurine [1, 2], a xanthine isomer evocatively termed yanthine [2]. This reaction is catalyzed by the *Clostridium sporogenes* XdhAC protein using an artificial electron donor [1] or by the ­combination of *Escherichia coli* XdhD and YgfM proteins using NADH as the reductant [2]. A subsequent reduction catalyzed by the NAD(P)H-dependent iron-sulfur dehydrogenase YgfK dearomatizes the purine ring to form dihydro-yanthine for the ring opening catalyzed by the aminohydrolase SsnA and formation of ureidomethyl-hydantoin (UMH) as the major product. Opening of the hydantoin ring catalyzed by the amidohydrolase HyuA produces 2,3-diureido propanoic acid (DUPA), an intermediate structurally reminiscent of allantoic acid in the aerobic pathway. Unlike allantoic acid, however, DUPA retains an aliphatic carbon that would otherwise be released as CO_2_ in the oxidative route.

At this stage, the proposed pathways diverge slightly (Fig. 1). According to Liu *et al.*, it is the 3-ureido group that undergoes cleavage by the carbamoyl transferase YgeW, forming 3-amino-2-ureido-propanoic acid [1], whereas according to Li *et al.*, the same enzyme acts on the 2-ureido group, thus forming 2-amino-3-ureido-propanoic acid (albizziin) [2]. This alpha-amino acid has been previously isolated from the seeds of the Persian silk tree (*Albizzia julibrissin*), a genus named after the Italian nobleman Filippo degli Albizzi. Hydrolysis of the remaining ureido group by YgeY in both cases produces 2,3-diaminopropanoic acid (DAP), which is converted by the pyridoxal phosphate-dependent ammonia-lyase YgeX into the final product, pyruvate.

How the pathway was discovered

Since the aerobic pathway for UA degradation was delineated more than 80 years ago—while some of its genes and enzymes were characterized only in more recent years—it may be surprising that this anaerobic counterpart has been uncovered only now. One explanation lies in their contrasting phylogenetic distributions: the aerobic pathway is widespread across prokaryotes and all eukaryotic kingdoms, whereas the anaerobic pathway is restricted to selected obligate or facultative anaerobic bacteria. These bacteria nevertheless account for more than one-fifth of culturable gut species, including the well-studied gut resident *Escherichia. coli*; yet, the ability of *E. coli* strains to degrade UA under laboratory conditions depends critically on the composition of the growth medium [4]. Another explanation lies in the intrinsic challenge of investigating redox-sensitive reactions under anaerobic conditions, which demands experimental setups not routinely available in every biochemistry laboratory.

To tell the truth, a distinct pathway for anaerobic purine ­metabolism had already been outlined in the 1950s by Jesse C. Rabinowitz through his pioneering studies on obligate purinolytic soil bacteria. However, the genes supporting that pathway, recently identified by Tong *et al.* through comparative genomics and experimental analyses, are found mainly in environmental species and are rare in the gut microbiota [3]. A decisive clue for the identification of the gut pathway came from RNA-seq differential expression profiling, revealing a set of UA-induced genes (*ygeX*, *ygeY*, *ygeW*, *ygfK*, and *ssnA*) shared by phylogenetically distant gut bacteria [4]. As often observed in bacteria, functionally related genes are nicely organized in compact genomic clusters, allowing the identification of additional pathway components, such as *xdhD* and *ygfM*, based on their proximity to already implicated genes. The colocalization of genes encoding membrane transporters, in this case the UA-specific permease UacT [7], provides further evidence for the functional role of the cluster. With the exception of *E. coli* YgeX, whose catalytic activity, though not its physiological role, had been characterized as diaminopropionate ammonia-lyase [8], none of the pathway components had been previously studied. Nevertheless, bioinformatics analyses provided preliminary clues to their possible molecular functions, such as oxidoreductase, amidohydrolase, or carbamoyltransferase, based on conserved protein domains or homology with characterized enzymes from the ­anaerobic pyrimidine degradation pathway.

Although bioinformatics can suggest potential pathway components and even outline plausible reactions [2], the actual sequence of biochemical transformations can only be established experimentally. No current computational method, for example, could have predicted the formation of the specific xanthine isomer generated by UA reduction catalyzed by a xanthine dehydrogenase homolog in the first step of the pathway. To resolve the complete uricolytic sequence, Liu *et al.* first inferred the reaction sequences from the accumulation of labeled intermediates in *C. sporogenes* through genetic knockouts and ^1^³C-label metabolomics [1]; *in vitro* assays with recombinant enzymes then confirmed individual reactions and their cofactor dependencies [1]. Li *et al.* adopted a straightforward biochemical approach, using purified enzymes from *E. coli* and *C. difficile* to rebuild the pathway *in vitro* and demonstrate catalytic activities step by step [2].

In both studies, a critical requirement has been the production of a functional molybdenum-dependent reductase catalyzing the initiating reaction. The *C. sporogenes* enzyme (XdhAC) was obtained as a chromosomally His-tagged protein generated by CRISPR editing, allowing native cofactor assembly [1]. The *E. coli* counterpart (XdhD) was expressed from a low-copy plasmid to avoid overloading the cofactor biosynthesis machinery and recovered as an active XdhD–YgfM complex through co-purification of the endogenous flavin adenine dinucleotide (FAD)-binding subunit, which provides the electron-transfer module [2]. Interestingly, a heterodimeric association between XdhD and YgfM, which are encoded by adjacent genes in the *E. coli* genomes, is predicted with very high confidence (ipTM = 0.94) by AlphaFold3, supporting the existence of a stable functional complex.

## Open questions and future directions

As with any major discovery, the elucidation of the reductive uricolytic pathway expands fundamental understanding but also raises new questions. Some of these questions are related to the chemistry and enzymology of the pathway itself. The exact sequence of ureido cleavage reactions remains to be established, particularly whether the key intermediate corresponds to albizziin or 3-amino-2-ureidopropanoate. The physiological electron donor (and possibly an accessory protein) of the initiating molybdenum enzyme, XdhAC, has yet to be discovered. Another open issue concerns stereochemistry. The R configuration of the chiral center in the ureido intermediates has been proposed [1], but the enzymatic step responsible for this stereochemical outcome is not yet clear. If confirmed, this stereochemistry would imply the D configuration for bacterial albizziin, opposite to the l-stereoisomer described for the plant amino acid. Furthermore, kinetic parameters for the ­pathway enzymes remain to be determined. Detailed steady-state ­kinetics will be relevant to identify the rate-limiting transformations and integrate this reductive pathway into broader models of microbial and host purine metabolism.

Other questions concern the evolution of this pathway. The anaerobic route clearly confers an advantage, as it allows bacteria to recycle purine carbon and nitrogen and produces ATP to sustain bacterial growth. The pathway appears to be highly specific for UA, as neither other purines (unless converted to UA) nor the downstream intermediates of the aerobic degradation pathway can enter it. One of these intermediates, allantoin—the end product of purine catabolism in most placental mammals (Fig. 1)—is instead degraded by *E. coli* through a dedicated catabolic route that shares some enzymes with the aerobic pathway [9]. Thus, the anaerobic route likely confers a selective advantage to bacteria inhabiting the gut of host species that have lost a functional uricase (Uox) and excrete UA as the end product of purine metabolism, such as many arthropods, reptiles, birds, and certain mammals. Among mammals, loss of Uox has so far been documented in apes, including humans, and in Tethytheria, including elephants.

An interesting question is whether differences in host purine metabolism are mirrored by differences in microbiota composition, and whether such differences could in turn have influenced the evolutionary loss of urate degradation pathways in hominoids and other animal lineages. Another intriguing aspect concerns the origin of the gut pathway: was it an ancient metabolic innovation later co-opted by gut bacteria to exploit a carbon- and nitrogen-rich substrate excreted by urate-producing hosts, or a more recent adaptation driven by colonization of the intestinal niche? The profound diversity between the pathways found in gut bacteria and environmental bacteria specialized in UA degradation [2, 3] seems to support the latter hypothesis.

## Intestinal uricolysis and its therapeutic potential

Intestinal UA elimination offers a safer alternative to renal excretion, which carries a risk of urate precipitation and crystal formation leading to nephropathy and renal failure. This vulnerability has recently re-emerged in the context of kidney xenotransplantation, where marked hyperuricosuria raises concern for urate nephropathy [10, 11]. Yet the same condition highlights a broader physiological limitation: the kidney alone provides a narrow margin for urate disposal. Harnessing the gut pathway by selective stimulation of uricolytic bacteria, or the administration of defined probiotic strains capable of degrading UA, could enhance intestinal clearance and reduce systemic urate levels without burdening the kidneys.

The *in vivo* evidence presented across the two studies demonstrates that the reductive uricolytic pathway shapes both microbial fitness and host urate homeostasis. In a gnotobiotic mouse model rendered hyperuricemic by dietary UA and oxonic acid, co-colonization experiments revealed a clear ecological advantage for uricolytic bacteria: within 1 week, the *C. sporogenes* strain carrying a deletion of the initiating reductase gene was almost completely displaced by its wild-type counterpart, which maintained stable colonization [1]. This competitive outcome highlights how host-derived urate creates a selective niche for bacteria capable of its reductive degradation and how such microbes, in turn, contribute to luminal urate clearance.

In a uricase-deficient (*Uox*^−/−^) mouse model of hyperuricemia, oral administration of an engineered *E. coli* Nissle 1917 strain constitutively expressing the complete uricolytic gene cluster (CBT2.0) leads to a sustained reduction in circulating urate together with significant decreases in plasma urea nitrogen and creatinine [2]. Histological analyses show markedly attenuated tubular injury and inflammation in the kidneys of treated mice. The engineered strain persists in the colon even after treatment withdrawal, and its abundance is inversely correlated with plasma urate, indicating durable colonization and continuous activity *in vivo*.

Finally, the identification of the first intermediate of the pathway as a detectable serum metabolite in humans adds translational significance: a preliminary LC–MRM–MS analysis revealed significantly higher yanthine concentrations in patients with gout than in healthy controls, suggesting that bacterial uricolysis contributes to the circulating purine pool and its products could serve as biomarkers of altered urate metabolism [2].

The elucidation of a bacterial reductive pathway for UA degradation underscores the role of the gut microbiota in systemic urate homeostasis. This anaerobic route, dependent on several cofactors, is widespread among commensal bacteria and may account for a substantial fraction of intestinal urate elimination, suggesting that micronutrient availability could influence host urate levels. Experimental reconstitution of the pathway and its transfer into a probiotic background demonstrated that intestinal uricolysis can be enhanced to lower serum urate and protect against urate-induced kidney injury *in vivo*. These findings highlight the gut microbiome as a modifiable component of urate metabolism and open new perspectives for microbiome-targeted interventions against hyperuricemia and gout.
